# Supplementary material for: A fault-intrusion-tolerant system and deadline-aware algorithm for scheduling scientific workflow in the cloud
Source: PeerJ Comput Sci. 2021 Nov 2;7:e747. doi: 10.7717/peerj-cs.747 (PMC8576554; doi:10.7717/peerj-cs.747)
Supplement: Supplemental Information 6 [file peerj-cs-07-747-s006.zip › Myproject1.1/docs/org/cloudbus/cloudsim/VmAllocationPolicySimple.html]

VmAllocationPolicySimple


JavaScript is disabled on your browser.


- Overview
- Package
- Class
- Use
- Tree
- Deprecated
- Index
- Help

- Prev Class
- Next Class

- Frames
- No Frames

- All Classes

- Summary:
- Nested |
- Field |
- Constr |
- Method

- Detail:
- Field |
- Constr |
- Method


org.cloudbus.cloudsim

## Class VmAllocationPolicySimple

- java.lang.Object
- - org.cloudbus.cloudsim.VmAllocationPolicy
  - - org.cloudbus.cloudsim.VmAllocationPolicySimple

- ---

    

  ```
  public class VmAllocationPolicySimple
  extends VmAllocationPolicy
  ```

  VmAllocationPolicySimple is an VmAllocationPolicy that chooses, as the host for a VM, the host
  with less PEs in use.

  Since:
  :   CloudSim Toolkit 1.0

- - ### Constructor Summary

    Constructors

    Constructor and Description

    `VmAllocationPolicySimple(java.util.List<? extends Host> list)`

    Creates the new VmAllocationPolicySimple object.
  - ### Method Summary

    Methods

    | Modifier and Type | Method and Description |
    | --- | --- |
    | `boolean` | `allocateHostForVm(Vm vm)` Allocates a host for a given VM. |
    | `boolean` | `allocateHostForVm(Vm vm, Host host)` Allocates a specified host for a given VM. |
    | `void` | `deallocateHostForVm(Vm vm)` Releases the host used by a VM. |
    | `protected java.util.List<java.lang.Integer>` | `getFreePes()` Gets the free pes. |
    | `Host` | `getHost(int vmId, int userId)` Gets the host that is executing the given VM belonging to the given user. |
    | `Host` | `getHost(Vm vm)` Gets the host that is executing the given VM belonging to the given user. |
    | `protected java.util.Map<java.lang.String,java.lang.Integer>` | `getUsedPes()` Gets the used pes. |
    | `java.util.Map<java.lang.String,Host>` | `getVmTable()` Gets the vm table. |
    | `java.util.List<java.util.Map<java.lang.String,java.lang.Object>>` | `optimizeAllocation(java.util.List<? extends Vm> vmList)` Optimize allocation of the VMs according to current utilization. |
    | `protected void` | `setFreePes(java.util.List<java.lang.Integer> freePes)` Sets the free pes. |
    | `protected void` | `setUsedPes(java.util.Map<java.lang.String,java.lang.Integer> usedPes)` Sets the used pes. |
    | `protected void` | `setVmTable(java.util.Map<java.lang.String,Host> vmTable)` Sets the vm table. |

    - ### Methods inherited from class org.cloudbus.cloudsim.VmAllocationPolicy

      `getHostList, setHostList`
    - ### Methods inherited from class java.lang.Object

      `clone, equals, finalize, getClass, hashCode, notify, notifyAll, toString, wait, wait, wait`

- - ### Constructor Detail


    - #### VmAllocationPolicySimple

      ```
      public VmAllocationPolicySimple(java.util.List<? extends Host> list)
      ```

      Creates the new VmAllocationPolicySimple object.

      Parameters:
      :   `list` - the list
  - ### Method Detail


    - #### allocateHostForVm

      ```
      public boolean allocateHostForVm(Vm vm)
      ```

      Allocates a host for a given VM.

      **Specified by:**
      :   `allocateHostForVm` in class `VmAllocationPolicy`

      Parameters:
      :   `vm` - VM specification

      Returns:
      :   $true if the host could be allocated; $false otherwise


    - #### deallocateHostForVm

      ```
      public void deallocateHostForVm(Vm vm)
      ```

      Releases the host used by a VM.

      **Specified by:**
      :   `deallocateHostForVm` in class `VmAllocationPolicy`

      Parameters:
      :   `vm` - the vm


    - #### getHost

      ```
      public Host getHost(Vm vm)
      ```

      Gets the host that is executing the given VM belonging to the given user.

      **Specified by:**
      :   `getHost` in class `VmAllocationPolicy`

      Parameters:
      :   `vm` - the vm

      Returns:
      :   the Host with the given vmID and userID; $null if not found


    - #### getHost

      ```
      public Host getHost(int vmId,
                 int userId)
      ```

      Gets the host that is executing the given VM belonging to the given user.

      **Specified by:**
      :   `getHost` in class `VmAllocationPolicy`

      Parameters:
      :   `vmId` - the vm id
      :   `userId` - the user id

      Returns:
      :   the Host with the given vmID and userID; $null if not found


    - #### getVmTable

      ```
      public java.util.Map<java.lang.String,Host> getVmTable()
      ```

      Gets the vm table.

      Returns:
      :   the vm table


    - #### setVmTable

      ```
      protected void setVmTable(java.util.Map<java.lang.String,Host> vmTable)
      ```

      Sets the vm table.

      Parameters:
      :   `vmTable` - the vm table


    - #### getUsedPes

      ```
      protected java.util.Map<java.lang.String,java.lang.Integer> getUsedPes()
      ```

      Gets the used pes.

      Returns:
      :   the used pes


    - #### setUsedPes

      ```
      protected void setUsedPes(java.util.Map<java.lang.String,java.lang.Integer> usedPes)
      ```

      Sets the used pes.

      Parameters:
      :   `usedPes` - the used pes


    - #### getFreePes

      ```
      protected java.util.List<java.lang.Integer> getFreePes()
      ```

      Gets the free pes.

      Returns:
      :   the free pes


    - #### setFreePes

      ```
      protected void setFreePes(java.util.List<java.lang.Integer> freePes)
      ```

      Sets the free pes.

      Parameters:
      :   `freePes` - the new free pes


    - #### optimizeAllocation

      ```
      public java.util.List<java.util.Map<java.lang.String,java.lang.Object>> optimizeAllocation(java.util.List<? extends Vm> vmList)
      ```

      **Description copied from class: `VmAllocationPolicy`**

      Optimize allocation of the VMs according to current utilization.

      **Specified by:**
      :   `optimizeAllocation` in class `VmAllocationPolicy`

      Parameters:
      :   `vmList` - the vm list

      Returns:
      :   the array list< hash map< string, object>>


    - #### allocateHostForVm

      ```
      public boolean allocateHostForVm(Vm vm,
                              Host host)
      ```

      **Description copied from class: `VmAllocationPolicy`**

      Allocates a specified host for a given VM.

      **Specified by:**
      :   `allocateHostForVm` in class `VmAllocationPolicy`

      Parameters:
      :   `vm` - virtual machine which the host is reserved to

      Returns:
      :   $true if the host could be allocated; $false otherwise


- Overview
- Package
- Class
- Use
- Tree
- Deprecated
- Index
- Help

- Prev Class
- Next Class

- Frames
- No Frames

- All Classes

- Summary:
- Nested |
- Field |
- Constr |
- Method

- Detail:
- Field |
- Constr |
- Method
